# Cost-Utility of a Two-Dose Human Papillomavirus Vaccination Programme Added to Cervical Cancer Screening Compared with Cervical Cancer Screening Alone in Korea

**DOI:** 10.31557/APJCP.2019.20.2.425

**Published:** 2019

**Authors:** Hyunju Lee, Sooyoung Hur, Hyeongap Jang, I-Heng Lee, Woo Yun Sohn, Georges Van Kriekinge, Kriekinge Kim

**Affiliations:** 1 *Seoul National University Bundang Hospital, Seoul National University College of Medicine, 82 Gumi-ro 173 Beon-gil, Bundang-gu, 463-707 Seongnam,*; 2 *Seoul St. Mary's Hospital, The Catholic University of Korea, 505 Banpo-dong, Seocho-gu, *; 3 *GSK, 9th Floor, LS Yong-san Tower, 92 Hangangdae-ro, Yongsan-gu,*; 7 *Samsung Medical Center, Sungkyunkwan University School of Medicine, 81 Irwon-ro Gangnam-gu, Seoul, Korea, *; 4 *GSK, 23 Rochester Park, Singapore 139234, Singapore,*; 5 *Present address, Gilead Sciences, Hong Kong, China,*; 6 *GSK, 20 Avenue Fleming, 1300 Wavre, Belgium.*

**Keywords:** Human papillomavirus, vaccine, cost-utility, Korea

## Abstract

**Background:**

Cervical cancer is caused by the human papillomavirus and is a leading cause of cancer death among young Korean women. Current screening programmes could benefit from the addition of HPV vaccination into their schedule to help reduce disease burden. Two-dose vaccination schedules targeting HPV types 16 and 18, which are responsible for most cervical cancer cases, have recently been approved. Of the two available vaccines, AS04-adjuvanted HPV16/18 vaccine (AS04-HPV16/18v) provides greater protection against non-vaccine oncogenic HPV, while HPV-6/11/16/18 vaccine (4vHPVv) provides protection against genital warts.

**Methods:**

The health and economic consequences of introducing a two-dose HPV vaccination programme in 12-year-old girls together with screening were assessed in the Korean healthcare setting using a previously-published Markov model.

**Results:**

Compared with screening alone, AS04-HPV16/18v was cost-effective (incremental cost-effectiveness ratio below and within the Korean Won [KRW] 20-30 million treshold). When comparing the two vaccines, at 3% discount rate, AS04-HPV16/18v dominated 4vHPVv (i.e., provided 174 more quality-adjusted life-years (QALYs), 304 more life-years (LYs) and cost-savings of KRW 980 million). At a 5% discount rate, AS04-HPV16/18v provided comparable QALYs (albeit 5 fewer), 105 more LYs and cost-savings of KRW 292 million compared with 4vHPVv. Results were particularly sensitive to the discount rate used, as the health benefits of preventing cervical cancer are observed much later than those of preventing genital warts.

**Conclusion:**

For the Korean setting, HPV vaccination with a two-dose schedule is a cost-effective option, and AS04-HPV16/18v is likely to offer better health outcomes at a cost-saving compared with 4vHPVv.

## Introduction

Cervical cancer (CC) is the 4^th^ most common cancer, and 3^rd^ leading cause of cancer death among Korean women aged 15 to 44 years (Korean Statistical Information Service (KOSIS), 2015). Around 70% of CC is caused by human papillomavirus (HPV) types 16 and 18 (Bruni et al., 2015), which vaccination could significantly reduce. A national screening programme for health insurance beneficiaries (since 1989) and Medicaid women (since 1999) (Konno et al., 2010) helps detect and hence treat precancerous cervical intraepithelial neoplasia (CIN) stages. A national HPV vaccination programme was launched in June 2016 for 12-year-old girls, using a two-dose schedule (Lee, 2016). Two available HPV vaccines were recently (2014) approved in a two-dose schedule (previously a three-dose schedule): AS04-adjuvanted HPV16/18 vaccine (*Cervarix*, GSK, Belgium; AS04-HPV16/18v) and HPV-6/11/16/18 vaccine (*Gardasil*, Merck and Co. Inc., USA; 4vHPVv) (Centers for Disease Control and Prevention, 2016). Both vaccines target HPV-16/18 types, and, 4vHPVv targets low-risk HPV types (6 and 11) causing genital warts (GW) and some grade 1 CIN (CIN1). Both vaccines also protect against oncogenic HPV-types not contained in the vaccine – so-called *cross-protection* (Brown et al., 2009; Kavanagh et al., 2017; Skinner et al., 2009; Tjalma et al., 2009; Woestenberg et al., 2015). Clinical trials report higher cross-protective efficacy with AS04-HPV16/18v, against non-vaccine oncogenic HPV types, compared with 4vHPVv (Di Mario et al., 2015; World Health Organization (WHO), 2014). Recent reports from population-based vaccination programmes have reported high overall vaccine efficacy against grade 2 and 3 CIN (CIN2/3) for AS04-HPV16/18v as well as significant cross-protection against HPV-31/33/45 which seem to confirm the findings of the clinical trials (BEG, 2017; Brotherton and Bloem, 2018; Cameron et al., 2017; Kavanagh et al., 2017; Woestenberg et al., 2018).

Health economic analyses are increasingly important within the Asia-Pacific region to inform vaccine policy decision-making, particularly as competing priorities for universal mass vaccination (UMV) exist requiring substantial annual investment. Economic assessments must reflect local disease epidemiology, treatment practices and costs. HPV vaccination with a three-dose schedule was estimated to be cost-effective in several studies in Asia (Demarteau and Standaert, 2010; Liu et al., 2010), except one (National Evidence-Based Healthcare Collaborating Agency (NECA), 2012). However, no studies have yet assessed the two-dose schedule in Korea. The World Health Organization (WHO) recommends to add HPV vaccination to existing cervical cancer screening, provided the introduction is programatically feasible, that sustained financing can be secured and cost-effectiveness of vaccination is considered (National Evidence-Based Healthcare Collaborating Agency (NECA), 2012; World Health Organization (WHO), 2014).

This study evaluated the epidemiological and economic consequences of introducing two-dose HPV UMV for 12-year-old girls in Korea. As the WHO recommends to add HPV vaccination to existing screening, this model compared two-dose AS04-HPV16/18v plus screening with two-dose 4vHPVv plus screening and with current screening alone. 

## Materials and Methods

*Model description*

A previously published Markov model was adapted (Demarteau and Standaert, 2010) to the Korean setting to assess lifetime costs and benefits of HPV vaccination scenarios, accounting for both oncogenic and low-risk HPV infections. This type of model (static, Markov process) was considered appropriate to meet the objective of this analysis as the model type adequately reproduces the long natural history of the disease. Dynamic transition models with extensive data-input would be difficult to parameterise in the context of Korea.

A cohort of 12-year-old girls (N=253,000 (Korean Statistical Information Service (KOSIS), 2015)) entered the model’s ‘No HPV’ state. The natural history from oncogenic HPV infection to invasive CC was represented by health states through which the cohort may progress, remain or regress. Subjects with CC may be cured or die of CC. This model also included a pathway from low-risk HPV infection to GW and CIN1. Detection of lesions depended on screening coverage and test sensitivity. Transition probabilities were calibrated to match the age-specific incidence of GWs, the incidence of CC and CC death rate in Korean women (National Evidence-Based Healthcare Collaborating Agency (NECA), 2012) (Supplementary Table 1). Pap-smear testing occurred every two years for women aged 30 to 74 years as per current government funding (Bruni et al., 2015). Screening coverage was 49.5% (Korean Statistical Information Service (KOSIS), 2015). Subjects with detected CIN stages had a reduced risk of progression and different treatment and follow-up costs. Although Korean guidelines recommend only follow-up of detected CIN1 cases, the model assumed a number cases received treatment, as advised by an expert panel based on real practice where elderly patients are likely to be treated rather than just followed up. 

The model ran for the cohort’s lifetime with up to 95 one-year cycles. Each health state had an associated cost and utility score. Values were summed at the end of the evaluation as accumulated values.

*Comparators and outcomes*

The model compared two-dose AS04-HPV16/18v plus screening with two-dose 4vHPVv plus screening and with screening alone. 

Predicted health outcomes included cases with invasive CC, CIN1, CIN2, CIN3 and GW, life-years (LY) and quality-adjusted life-years (QALY) gained. Incremental cost-effectiveness ratios (ICERs) were calculated; cost per LY and per QALY gained. The acceptable cost-effectiveness threshold was Korean Won (KRW) 20 to 30 million in Korea, according to the National Evidence-Based Healthcare Collaborating Agency (NECA) (National Evidence-Based Healthcare Collaborating Agency (NECA), 2012). 

Analyses were conducted from the Ministry of Health and Welfare perspective; direct costs included hospitalisation, screening tests and procedures and vaccines. As Korean patients contribute to healthcare costs via a co-payment system (e.g., drug cost via co-payment for listed drugs or an out-of-pocket payment for unlisted drugs), costs in the model combined the health insurance benefit and patient co-pay (National Evidence-Based Healthcare Collaborating Agency (NECA), 2012). This was in line with previous analyses undertaken in Korea (National Evidence-Based Healthcare Collaborating Agency (NECA), 2012).

*Inputs and Assumptions*

*1. Vaccination*

Both vaccination programmes assumed the same coverage, duration of protection and vaccine cost. Key differences regarded effectiveness against non-vaccine oncogenic HPV (i.e., cross protection) and GW prevention.

Coverage (86%) was based on other coverage rates achieved in the National Immunisation Programme (National Evidence-Based Healthcare Collaborating Agency (NECA), 2012). All vaccinated girls received two doses. Duration of protection was assumed lifelong against both vaccine (Aregay et al., 2013; Naud et al., 2014) and non-vaccine (Moscicki et al., 2015) HPV types in the base case.

Vaccine effectiveness included efficacy against vaccine types and cross-protection efficacy. Based on trial results, 98% vaccine efficacy (VE) was assumed for all vaccine-type HPV (i.e., HPV types 6/11/16/18) as per vaccine composition for both vaccines (Paavonen et al., 2009; The FUTURE II Study Group, 2007). However, cross-protective efficacy against HPV types 31/33/35/39/45/51/52/56/58/59 was differentiated based on the two vaccines’ respective trial efficacy (Brown et al., 2009; Paavonen et al., 2009; Skinner et al., 2009; The FUTURE II Study Group, 2007; Tjalma et al., 2009). Using Korean-specific distributions of HPV types for each lesion (Bruni et al., 2015), the proportion of each HPV type in the lesion was multiplied by the VE against the specific lesion types to approximate vaccine effectiveness (Table 1). 

Trial efficacy data were from HPV-naïve women for the relevant HPV type at study entry, to represent girls pre-sexual debut such as 12-year-old girls. 

Recent randomised trials assessing immunogenicity and safety of AS04-HPV16/18v in 9-14-year-old girls demonstrated the two-dose schedule to be non-inferior to the three-dose schedule in 15-25-year-old girls (Romanowski et al., 2016). Therefore, two-dose efficacy in 9-14-year-old girls was inferred by immunobridging from previous trials (Paavonen et al., 2009). For 4vHPVv, two-dose efficacy was assumed to be the same as three-dose, based on a Canadian study (Dobson et al., 2013). 

*2. Costs and utilities*

Both vaccines were assumed to cost KRW 105,000/dose (i.e., a 30% discount on current private setting patient costs). Treatment costs for CIN lesions, CC and GWs were from the Korean NECA report (2012), based on national health insurance claims from 2010 (National Evidence-Based Healthcare Collaborating Agency (NECA), 2012). The treatment cost for CC with and without recurrence was based on total costs for the first year and three consecutive years of treatment; and were considered lifetime costs of CC treatment. To fit the model’s annual cycles, lifetime cost was adjusted to the average time spent in the CC state (i.e., 2.5 years). Where local data were lacking, data from another Asian country were used. Data were validated by clinical experts with extensive experience of CC treatment.

Disutility weights for precancerous stages and CC were from published HPV cost-effectiveness analyses. No decrements in utility were assumed by age; instead, disutilities in Supplementary Table 1 were subtracted from a baseline utility of 1 across all ages for every year in the model.

*3. Discount rate and price conversion*

As per Korean guidelines, costs and outcomes were discounted at 5%/annum in the base-case (International Society for Pharmacoeconomics and Outcomes Research (ISPOR), 2010; National Evidence-Based Healthcare Collaborating Agency (NECA), 2012). Based on WHO and USA recommendations, a 3% discount rate was also evaluated (Academy of Managed Care Pharmacy (AMCP), 2012; Walker et al., 2010). Costs were updated to 2014 values using the Korean consumer price index for health (Korean Statistical Information Service (KOSIS), 2015).

*Scenario and sensitivity analyses*

A first scenario assessed alternative discount rates. As the time to achieve benefits is much greater for CC prevention than for GW prevention, the discount rate has an impact on the relative effect of the two vaccines. Recent guidelines by the UK National Institute for Health and Care Excellence (NICE) recommend using a 1.5% discount rate when benefits of the intervention are of long duration, such as 30 years or lifetime (National Institute for Health and Care Excellence (NICE), 2013). In this scenario, a 1.5% discount rate was applied to costs and outcomes related to CC and GWs alike. 

A second scenario assessed the prevention of oncogenic HPV alone. Efficacy against low-risk HPV was set to 0% for both vaccines. 

A third scenario varied duration of cross-protection efficacy between 10 and 50 years. In this scenario, 40% of the cohort received a booster dose at the time of waning of VE. An alternate variation of this scenario was conducted assuming 0% booster doses would be given.

One-way sensitivity analyses evaluated how robust results were to changes in model variables, using 95% confidence intervals for cross-protection efficacy and ± 20% of base-case values for other input parameters (Table 1). 

**Figure 1:**
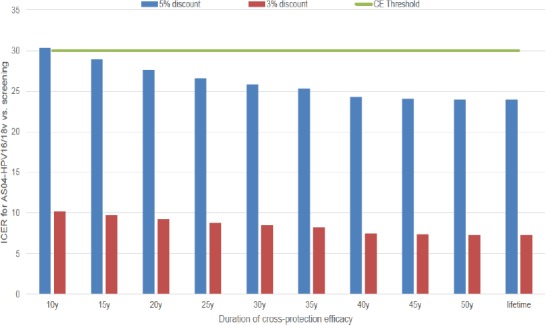
Impact of Varying Duration of Cross Protection Efficacy of AS04-HPV16/18v + screening vs. Screening Alone on ICER (M, KRW). AS04-HPV16/18v, AS04-adjuvanted HPV16/18 vaccine; CE, cost-effectiveness; ICER, incremental cost-effectiveness ratio; KRW, Korean Won; M, million; y, years

**Figure 2:**
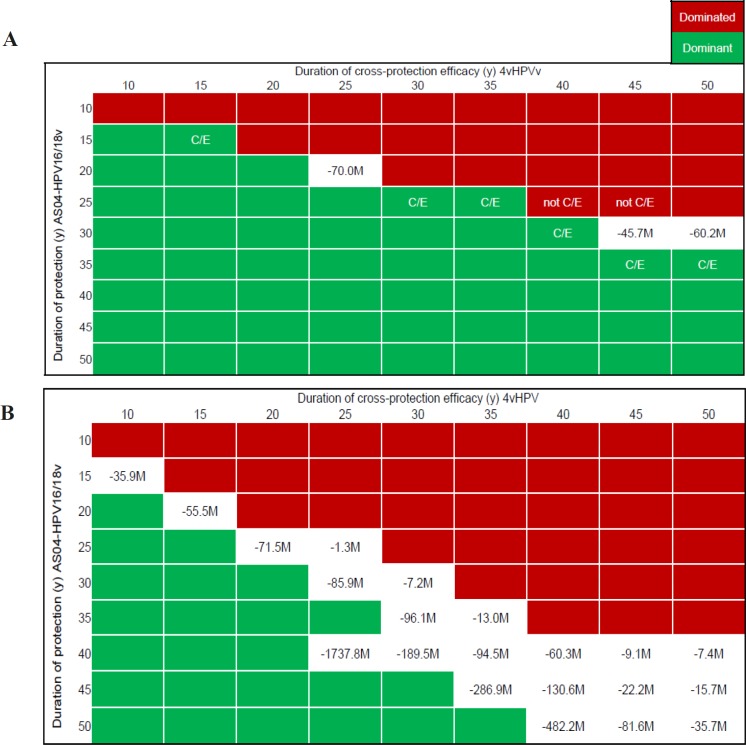
Impact of Cross-Protection Efficacy Duration and Discount Rate on ICER. * Negative ICER occurred when cost savings occur for a loss of QALY. 4vHPVv, HPV-6/11/16/18 vaccine; AS04-HPV16/18v, AS04-adjuvanted HPV16/18 vaccine; C/E, cost-effective; ICER, incremental cost-effectiveness ratio; M, million; y, years

**Figure 3:**
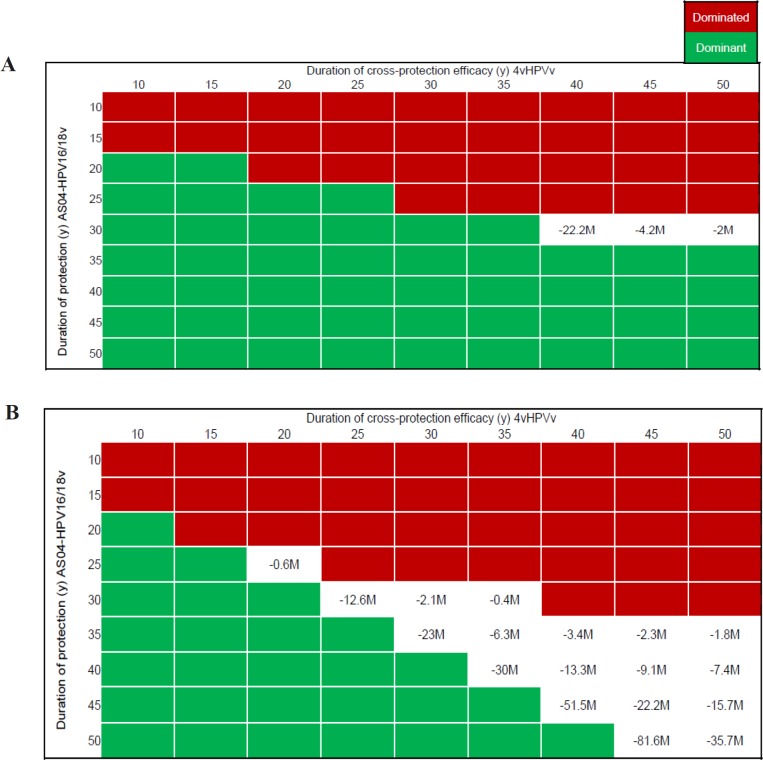
Impact of Cross-Protection Efficacy Duration and Discount Rate on ICER Assuming no Booster Dose. 4vHPVv, HPV-6/11/16/18 vaccine; AS04-HPV16/18v, AS04-adjuvanted HPV16/18 vaccine; ICER, incremental cost-effectiveness ratio; M, million; y, years

**Figure 4:**
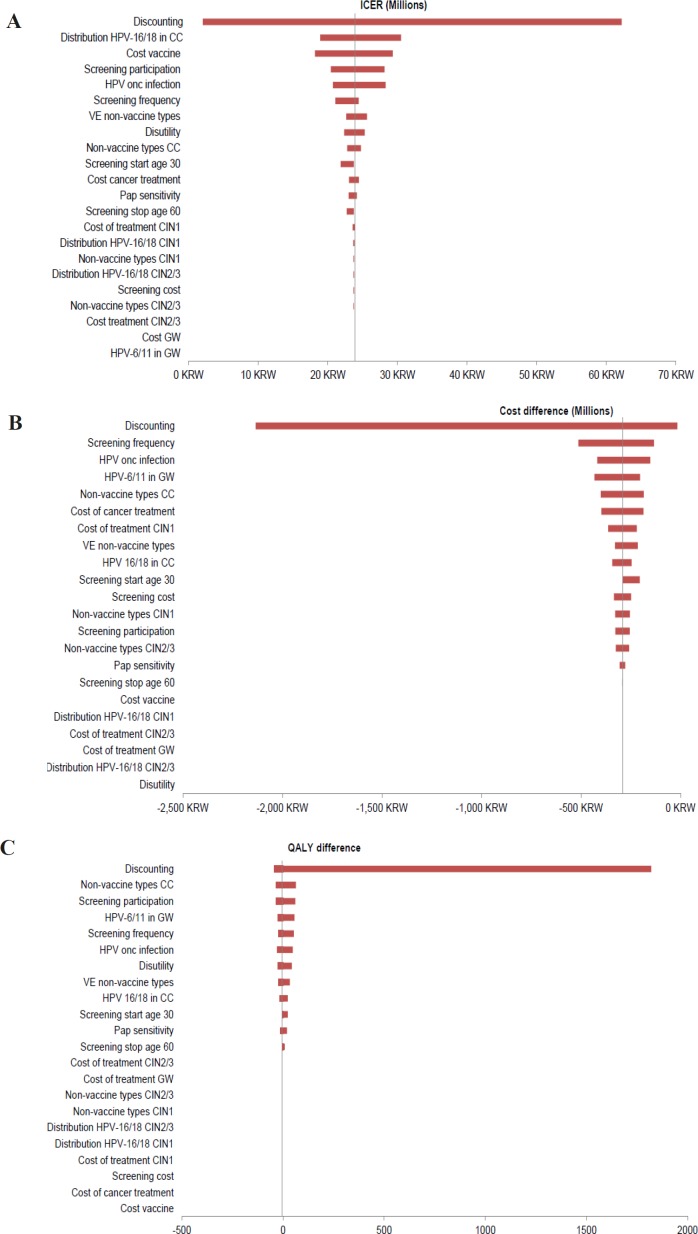
One-Way Sensitivity Analyses: Impact on ICER. 4vHPVv, HPV-6/11/16/18 vaccine; AS04-HPV16/18v, AS04-adjuvanted HPV16/18 vaccine; CC, cervical cancer; CIN, cervical intraepithelial neoplasia; GW, genital warts; HPV, human papillomavirus; ICER, incremental cost-effectiveness ratio; onc, oncogenic; QALY, quality-adjusted life-year; VE, vaccine efficacy

**Table 1:** HPV Type Distribution and Vaccine Effectiveness by Health State

Parameter	HPV type distribution	AS04-HPV16/18v efficacy (95%CI)	4vHPVv efficacy (95%CI)
CIN1			
HPV-16/18	33.2% (Bruni et al., 2015)	98% (Paavonen et al., 2009)	98% (The FUTURE II Study Group, 2007)
Cross protection	39.2% (Bruni et al., 2015)	48% (28.9 - 61.9) (Paavonen et al., 2009; Tjalma et al., 2009)	23% (7.8 - 36.4) (Brown et al., 2009)
HPV-6/11	1.7% (Bruni et al., 2015)	0%	98% (The FUTURE II Study Group, 2007)
Overall effectiveness		51.2%	43.2%
CIN2/3			
HPV-16/18	40.6% (Bruni et al., 2015)	98% (Paavonen et al., 2009)	98% (The FUTURE II Study Group, 2007)
Cross protection	52.6% (Bruni et al., 2015)	68% (45.7 - 82.4) (Paavonen et al., 2009; Skinner et al., 2009)	33% (6.0 - 51.9) (Brown et al., 2009)
Overall effectiveness		75.8%	57.1%
Cervical Cancer			
HPV-16/18	70.3% (Bruni et al., 2015)	98% (Paavonen et al., 2009)	98% (The FUTURE II Study Group, 2007)
Cross protection	17.7% (Bruni et al., 2015)	68% (45.7 - 82.4) (Paavonen et al., 2009; Skinner et al., 2009)	33% (6.0 - 51.9) (Brown et al., 2009)
Overall effectiveness		81.0%	74.7%
Genital warts			
HPV-6/11	90.0%^a^)	0%	98% (The FUTURE II Study Group, 2007)
Overall effectiveness		0%	88.2%

^a)^, Expert opinion; Cross-protection: based on HPV types 31, 33, 35, 39, 45, 51, 52, 56, 58 and 59; 4vHPVv: HPV-6/11/16/18 vaccine; 95% CI, 95% confidence interval; AS04-HPV16/18v, AS04-adjuvanted HPV16/18 vaccine; CIN, cervical intraepithelial neoplasia; HPV, Human Papillomavirus

**Table 2:** Predicted Lifetime Outcomes, Costs (2014 KRW) and Incremental Results

	Screening alone	Screening + AS04-HPV16/18v	Screening + 4vHPVv	
Number of cases, LYs and QALYs (undiscounted)				
CIN1 cases detected	37,127	24,868	27,644	
CIN2/3 cases detected	4,349	1,844	2,562	
CC cases	4,081	1,528	1,767	
CC deaths	1,641	614	710	
GW cases	14,955	14,955	4,273	
LYs	18,516,397	18,535,824	18,534,000	
QALYs	18,508,029	18,532,268	18,530,493	
Cost breakdown (undiscounted)				
Vaccination	0	45,691,800,000	45,691,800,000	
Screening	109,741,428,365	111,406,081,748	112,388,154,487	
CIN1 treatment	12,044,348,097	7,956,977,842	8,850,502,725	
CIN2/3 treatment	4,565,154,479	1,913,419,835	2,666,602,622	
GW treatment	2,775,475,118	2,775,475,118	793,078,196	
CC treatment	66,655,820,205	24,955,824,281	28,858,463,415	
Total direct costs	195,782,226,264	194,699,578,824	199,248,601,445	
Cost breakdown (discounted 5%)				
Vaccination	0	45,691,800,000	45,691,800,000	
Screening	19,246,198,646	19,581,894,693	19,798,211,668	
CIN1 treatment	2,573,266,040	1,737,524,635	1,927,362,247	
CIN2/3 treatment	1,026,220,848	429,630,917	598,865,275	
GW treatment	1,135,099,252	1,135,142,772	322,480,878	
CC treatment	9,088,653,737	3,343,649,136	3,872,840,707	
Total direct costs	33,069,438,523	71,919,642,153	72,211,560,775	
LYs	5,120,917	5,122,038	5,121,933	
QALYs	5,119,899	5,121,525	5,121,530	
Cost breakdown (discounted 3%)				
Vaccination	0	45,691,800,000	45,691,800,000	
Screening	36,522,795,018	37,131,591,147	37,511,513,733	
CIN1 treatment	4,573,562,170	3,063,918,165	3,402,596,208	
CIN2/3 treatment	1,782,619,194	747,653,613	1,041,717,551	
GW treatment	1,578,789,711	1,578,874,638	449,880,717	
CC treatment	18,776,321,142	6,968,344,768	8,064,716,482	
Total direct costs	63,234,087,235	95,182,182,331	96,162,224,691	
LYs	7,608,185	7,611,441	7,611,137	
QALYs	7,606,037	7,610,437	7,610,263	
Incremental results	AS04-HPV16/18v vs. screening	AS04-HPV16/18v vs. 4vHPVv		
Base-case, 5% discounted				
Incremental costs	38,850,203,630	-291,918,622		
LY gained	1,121	105		
QALYs gained	1,626	-5		
Cost per QALY gained	23,893,114	Negative ICER^a^): -58,383,724		
Base-case, 3% discounted				
Incremental costs	31,948,095,096	-980,042,360		
LY gained	3,256	304		
QALYs gained	4,400	174		
Cost per QALY gained	7,260,931	AS04-HPV-16/18v dominates		
Incremental results	AS04-HPV16/18v vs. screening	AS04-HPV16/18v vs. 4vHPVv	
Scenario, 1.5% discounted			
Incremental costs	21,060,578,273	-2,135,253,813	
LYs gained	7,740	726	
QALYs gained	10,007	599	
Cost per QALY gained	2,104,585	AS04-HPV-16/18v dominates	
Scenario Oncogenic HPV, 5% discounted			
Incremental costs	38,850,203,630	-857,668,791	
LY gained	1,121	105	
QALYs gained	1,626	151	
Cost per QALY gained	23,893,114	AS04-HPV-16/18v dominates	
Scenario Oncogenic HPV, 3% discounted			
Incremental costs	31,948,095,096	-1,676,959,893	
LY gained	3,256	304	
QALYs gained	4,400	411	
Cost per QALY gained	7,260,931	AS04-HPV-16/18v dominates	

^a^, Negative ICER: fewer QALYs gained at a cost-saving; 4vHPVv, HPV-6/11/16/18 vaccine; AS04-HPV16/18v, AS04-adjuvanted HPV16/18 vaccine; CC, cervical cancer; CIN, cervical intraepithelial neoplasia; GW, genital warts; KRW, Korean Won; LY, life-year; QALY, quality-adjusted life-year.

## Results

*Model validation*

The modelled estimates were a close approximation to the observed incidence of CC and GW in Korea as well as CC mortality before vaccination (Supplementary Figures 1, 2, 3).

*Base-case results*

Introducing two-dose mass HPV vaccination in 12-year-old girls compared with screening alone was shown to significantly reduce detected precancerous CIN cases, detected CC cases and deaths from CC (AS04-HPV16/18v and 4vHPVv), as well as GW cases (4vHPVv). 

AS04-HPV16/18v was predicted to prevent more CIN1, CIN2/3, CC cases and CC deaths than 4vHPVv. AS04-HPV16/18v prevented 14,764 precancerous cases, 2,553 CC cases and 1,027 CC deaths versus screening alone. Versus screening alone, 4vHPVv prevented 10,682 GW cases. As a result, the model predicted a gain in LYs and QALYs with vaccination programmes versus screening alone (Table 2). Overall QALYs associated with both vaccines were fairly comparable, although they were driven by greater prevention of oncogenic disease in the case of AS04-HPV16/18v (i.e., 3,494 more precancerous cases, 239 more CC cases and 96 more CC deaths prevented than with 4vHPVv), and driven by lower prevention of oncogenic disease plus prevention of GWs (10,682 GW cases prevented) in the case of 4vHPVv. 

Compared with screening alone, implementing a vaccination programme was predicted to result in increased undiscounted direct costs due to the vaccine cost but decreased undiscounted treatment costs in all disease states, due to disease prevention. The most significant cost saving was remaining CC treatment costs (KRW 25.0-28.9 billion; undiscounted) versus screening alone (KRW 66.7 billion; undiscounted). AS04-HPV16/18v was associated with lower treatment costs for all detected CIN stages (saving of KRW 1.6 billion, undiscounted) and CC (savings of KRW 3.9 billion, undiscounted) than 4vHPVv. Conversely, the costs of treating GWs were lower with 4vHPVv (saving of KRW 2.0 billion, undiscounted). The total direct costs were lower with AS40-HPV-16/18v than 4vHPVv (Table 2). 

*Incremental costs and outcomes*

Compared with screening alone, AS04-HPV16/18v resulted in increased (discounted) costs for an increase in (discounted) LYs and QALYs. The cost per QALY gained was KRW 23.9 million (5% discount rate) and KRW 7.3 million (3% discount rate). 

When comparing both vaccines, AS04-HPV16/18v was found to save more costs (KRW 291.9 to 980.0 million, 5% and 3% discount rate respectively), with a minor decrement in QALYs at the 5% discount rate (5 fewer QALYs) and a gain in 174 QALYs at the 3% discount rate. Therefore, at a 5% discount rate, AS04-HPV16/18v provided a fairly comparable (albeit slightly smaller) amount of benefit at a cost-saving. At a 3% discount rate, AS04-HPV16/18v dominated 4vHPVv (i.e., provided more health benefits at a cost-saving) (Table 2).

*Scenario analyses*

*1- Discount rate for oncogenic HPV-related benefits*

At a 1.5% discount rate for both costs and outcomes (due to the long duration to achieve CC benefits), AS04-HPV16/18v resulted in more QALYs gained compared with both screening alone and 4vHPVv. Despite vaccination costs, AS04-HPV16/18v was cost-effective versus screening alone, at a cost per QALY gained of KRW 2.1 million, well below the KRW 20-30 million threshold. Compared with 4vHPVv, AS04-HPV16/18v was dominant, with additional QALYs gained at a cost-saving (Table 2).

*2- Vaccine impact on oncogenic HPV*

When focussing on CC-related (oncogenic) HPV alone, AS04-HPV16/18v was dominant (i.e., more costs were saved and more QALYs were gained) over 4vHPVv, due to its greater protection against oncogenic HPV. The costs saved and QALYs gained were greater when using a 3% versus a 5% discount rate (KRW -1.7 billion vs. -857.7 million saved and 411 vs. 151 QALYs gained respectively) (Table 2).

*3- Duration of cross-protection efficacy*

When reducing cross-protection efficacy duration to 15 years and assuming a booster dose is given at the time of waning to 40% of the cohort, AS04-HPV16/18v remained cost-effective compared with screening alone at a 5% discount rate, but not at 10 years (threshold surpassed at cost per QALY gained of KRW 30.3 million). At 3% discount rate, AS04-HPV16/18v was always cost-effective compared with screening alone (Figure 1). 

Compared with 4vHPVv, reducing cross-protection efficacy duration (to 10 years for both vaccines) resulted in AS04-HPV16/18v being either dominant or resulting in a high negative ICER (indicating large cost savings for a small QALY loss) in the majority of cases (Figure 2). 

Assuming 4vHPVv had a longer duration of cross-protection efficacy (of 5 years or more) compared with AS04-HPV16/18v and that no booster dose would be given, the former tended to dominate or lead to high negative ICERs, especially at a 5% versus 3% discount rate. However, AS04-HPV16/18v remained dominant or cost-effective whenever cross-protection efficacy duration was 35 years or more (Figure 3). 

*Sensitivity analyses*

One-way sensitivity analyses on AS04-HPV16/18v versus screening alone found the ICER was most sensitive to the discount rate, followed by HPV-16/18 distribution in CC, vaccine cost and screening parameters (Figure 4a). 

One-way sensitivity analyses on the comparison between vaccines assessed cost and QALY impacts separately due to the negative ICER. Costs were mainly influenced by discount rate, screening frequency, rate of oncogenic HPV infection, HPV distribution-related parameters and cancer treatment cost (Figure 4b). For QALYs, discount rate was the most influential variable, followed by HPV distribution in CC and GWs, screening participation and HPV incidence (Figure 4c). 

## Discussion

The cost-effectiveness of adding vaccination to the national screening programme was explored, and found to reduce CC burden in Korea. 

The model predicted 4vHPVv would prevent GW cases compared with AS04-HPV16/18v, however AS04-HPV16/18v would prevent more CC cases and deaths, increasing LYs and QALYs versus 4vHPVv. Treatment cost savings due to GWs with 4vHPVv were offset by those due to CC prevention with AS04-HPV16/18v. 

AS04-HPV16/18v produced more health benefits at an additional cost compared with screening alone and would be a cost-effective option for Korea (ICER of KRW 7.3 million or 23.9 million, at 3% or 5% discount rate, respectively). At a 5% discount rate, despite more LYs at a cost saving, AS04-HPV16/18v provided a comparable amount of QALYs (namely a loss of 5 QALYs) versus 4vHPVv, resulting in a high negative ICER. AS04-HPV-16/18v was the dominant choice for HPV vaccination in the Korean context (QALY gain of 174, cost savings of 980.0 million) at a 3% discount rate. A larger discount rate reduced the value of the longer term benefits (CC prevention) achieved by AS04-HPV16/18v, and increased the value of shorter term GW benefits achieved with 4vHPVv. A lower discount rate is arguably more appropriate to model prevention of CC as these benefits occur much later in time. When using the NICE-recommended discount rate of 1.5% (National Institute for Health and Care Excellence (NICE), 2013), AS04-HVP-16/18v resulted in a much lower ICER versus screening alone (i.e., KRW 2.1 million), and, more than tripled the QALY benefit versus 4vHPVv (seen with 3% discount rate), thus remaining the dominant choice in the Korean context. Discount rate had a large effect on cost-effectiveness. 

Although this model assumed no effect on prevention of GWs with AS04-HPV16/18v, recent data from the UK observed a moderate cross-protective effect (Canvin et al., 2017). This was confirmed in a pivotal Phase III trial post-hoc analysis for AS04-HPV16/18v reporting a 34.5% cross-protection efficacy against 6-month persistent HPV 6 and 11 infections (Szarewski et al., 2013). This would increase the QALY gain, at all discount rates, further improving the cost per QALY ratio versus 4vHPVv. 

At least 15 years of cross-protection efficacy was needed for AS04-HPV16/18v to remain cost-effective versus screening alone. Compared with 4vHPVv, AS04-HPV16/18v was dominant or cost-effective in the majority of cases at a 3% discount rate, when reducing the duration of cross-protective efficacy. Clinical trial data suggest, however, that waning of cross-protection efficacy is unlikely, as antibody titres remained at a stable plateau after 9.4 years follow-up (Moscicki et al., 2015; Naud et al., 2014; Taylor et al., 2016). It is, however, unlikely that 4vHPVv will have a longer duration of cross-protection than AS04-HPV16/18v as head-to-head immunogenicity trials found the geometric mean titers (GMTs) of AS04-HPV16/18v were at least 7-8 fold higher than 4vHPVv up to 5 years after vaccination (Einstein et al., 2014). Additionally, population-based vaccination results have demonstrated cross-protection to be impactful up to 7 years post vaccination (BEG, 2017; Kavanagh et al., 2017; Woestenberg et al., 2018).

In one-way sensitivity analyses, discount rate and a lower distribution of HPV-16/18 in CC increased the ICER over the threshold for AS04-HPV16/18v versus screening alone. When comparing the two vaccines, discount rate had the biggest impact on cost and QALY difference. The effect and importance of discount rate has been recognised in several HPV models with a reminder to policy-makers to understand the implications of discount rates applied. 

These findings differ from the Korean Ministry of Health’s analysis (NECA report) which concluded HPV vaccination (using a 50/50 share of AS04-HPV16/18v and 4vHPVv vaccination) would not be cost-effective (ICER of KRW 32.4M/QALY) versus screening (National Evidence-Based Healthcare Collaborating Agency (NECA), 2012). This may be due to differences in model structure, transition probabilities and costs included. The present model predicted similar outcomes compared with the NECA analysis (data not shown) when using the NECA inputs. Both studies did not consider herd immunity effects and assumed lifelong protection. There were major differences, however; the NECA study did not include cross-protection benefits and used a conservative assumption for VE that did not account for vaccination of HPV-naïve individuals (i.e., 45% for those receiving at least one dose compared with 98% in the current analysis).

This analysis has limitations. A static Markov model was used which, unlike more complex dynamic models, does not account for herd protection benefits in unvaccinated people. These additional cases prevented, achieved without additional cost, can be expected to improve ICERs versus screening alone, as observed in HPV models with herd protection. In the current analysis, when comparing vaccination plus screening versus screening alone, accounting for herd protection could have therefor a more favourable ICER and most likely below the KRW 20,000,000 mark at a 5% discount rate. Required data around disease transmission, however, may be difficult to obtain. Also, all oncogenic HPV types and low-risk HPV types were considered together, not taking into account any differential progression or regression of specific HPV types.

AS04-HPV16/18v plus screening was predicted to avert more CC-related morbidity and mortality, due to additional cross-protection efficacy compared with 4vHPVv plus screening. More CIN and CC cases prevented resulted in greater cost savings than from GW prevention with 4vHPVv. AS04-HPV16/18v plus screening was cost-effective, and the dominant choice in most scenarios for a UMV programme in Korea.

## Trade mark section

Cervarix is a trade mark owned by or licensed to the GSK group of companies. Gardasil is a trade mark of Merck and Co. Inc.

## Authors’ contributions

All authors had full access to all of the data in the study and can take responsibility for the integrity of the data and the accuracy of the data analysis. SH, HL, IHL and GVK conceived and designed the study and were involved in the development of the model. The data were acquired and analysed by all the authors. All authors participated in the development of this manuscript and gave final approval before submission.

## Conflict of interest

WYS and GVK are employees of the GSK group of companies. HJ was an employee of the GSK group of companies at the time of the study. IHL was an employee of the GSK group of companies at the time of the study and is now an employee of Gilead Sciences, Hong Kong. BGK, HL and SH report personal fees from the GSK group of companies during the conduct of the study and outside the submitted work.
